# Switch from intravenous to subcutaneous immunoglobulin IgPro20 in CIDP patients: a prospective observational study under real-world conditions

**DOI:** 10.1177/17562864211009100

**Published:** 2021-04-16

**Authors:** Stefan Gingele, Moritz Koch, Anna Christina Saparilla, Gudrun M. Körner, Jarle von Hörsten, Marina Gingele, Tabea Seeliger, Franz Felix Konen, Martin W. Hümmert, Alexandra Neyazi, Martin Stangel, Thomas Skripuletz

**Affiliations:** Department of Neurology, Hannover Medical School, Hannover, Germany; Department of Neurology, Hannover Medical School, Hannover, Germany; Department of Neurology, Hannover Medical School, Hannover, Germany; Department of Neurology, Hannover Medical School, Hannover, Germany; Department of Neurology, Hannover Medical School, Hannover, Germany; Department of Neurology, Hannover Medical School, Hannover, Germany; Department of Human Genetics, Hannover Medical School, Hannover, Germany; Department of Neurology, Hannover Medical School, Hannover, Germany; Department of Neurology, Hannover Medical School, Hannover, Germany; Department of Neurology, Hannover Medical School, Hannover, Germany; Department of Psychiatry, Social Psychiatry and Psychotherapy, Hannover Medical School, Hannover, Germany; Department of Neurology, Hannover Medical School, Hannover, Germany; Department of Neurology, Hannover Medical School, Carl-Neuberg-Str. 1, Hannover 30625, Germany

**Keywords:** Chronic inflammatory demyelinating polyneuropathy, intravenous immunoglobulin, subcutaneous immunoglobulin

## Abstract

**Background:**

IgPro20 is the first approved subcutaneous immunoglobulin (SCIg) preparation for the treatment of patients with chronic inflammatory demyelinating polyneuropathy (CIDP). Two different doses of the SCIg preparation were investigated in the pivotal PATH study. Real-world data, and particularly the efficacy of an equivalent dose switch from intravenous immunoglobulin (IVIg) to SCIg, are still not available.

**Methods:**

In this prospective observational study, 41 patients with CIDP treated with intravenous immunoglobulin (IVIg) were changed to an equivalent (1:1) dose of IgPro20 1 week after last IVIg treatment. Patients were examined at the time of switch from IVIg to SCIg, after 3 and after 6 months and efficacy, treatment preferences and systemic and local reactions were assessed.

**Results:**

Various clinical outcome parameters demonstrated overall stability regarding disability, general activity and social participation, grip and muscle strength, as well as gait impairment. Treatment satisfaction remained unchanged between IVIg and SCIg therapy. However, 88% of patients favoured treatment with subcutaneous IgPro20 over IVIg 6 months after switch to IgPro20.

**Conclusion:**

Results demonstrate that the switch of IVIg to an equivalent dose of SCIg represents an effective and preferred treatment option for CIDP patients.

## Introduction

Chronic inflammatory demyelinating polyneuropathy (CIDP) is the most prevalent chronic immune-mediated polyneuropathy. Repeated administration of intravenous immunoglobulin (IVIg) is an effective and widely used first-line therapeutic option for patients with CIDP, with the majority of patients requiring long-term maintenance IVIg therapy.^1^ Subcutaneous immunoglobulin (SCIg) represents a promising alternative to IVIg since it has a favourable pharmacokinetic profile resulting in a reduction of systemic adverse events and prevention of low IgG trough levels with associated clinical wearing-off signs. In addition, SCIg therapy increases patient autonomy and quality of life and is therefore preferred by many patients.^1,2^ Different preparations of subcutaneous immunoglobulin have been shown effective and well tolerated in the treatment of CIDP in preselected patient cohorts.^3–5^ In the randomized, double-blind, placebo-controlled phase III PATH study, two different doses of the SCIg preparation IgPro20 (0.2 g/kg and 0.4 g/kg per week) were well tolerated and demonstrated efficacy in the treatment of CIDP patients previously treated with IVIg.^2^ Based on these results, IgPro20 has recently been approved for the maintenance treatment of patients with CIDP after stabilization with IVIg. Until now, real-world data and the efficacy of an equivalent dose switch from IVIg to SCIg are lacking. Therefore, we prospectively monitored 41 patients with CIDP during and after switch from IVIg to IgPro20 regarding efficacy and patient preferences.

## Patients and methods

### Patients

Between May 2018 and November 2019, 102 patients with CIDP treated with IVIg at the Department of Neurology of Hannover Medical School were offered a treatment change to subcutaneous IgPro20. Forty-one patients decided to switch from IVIg to SCIg. All patients fulfilled the criteria for definite or probable CIDP according to the European Federation of Neurological Societies/Peripheral Nerve Society 2010 criteria.^6^ All patients showed a stable clinical course under treatment with IVIg and were switched to an equivalent (1:1) dose of SCIg 1 week after last IVIg treatment. Therefore, the previous dose of IVIg was divided by the weeks of the interval between the repeated IVIg treatments to receive a weekly immunoglobulin dose which was then given as weekly dose of SCIg starting 1 week after last IVIg administration. Patients gave written informed consent before being included in this prospective observational study. This investigation was approved by the Ethics Committee of Hannover Medical School (no. 7335).

### Procedures

Patients were examined at four different time points: immediately before the last course of IVIg treatment (‘−1 week’; data not shown and not included in the analysis), 1 week after the last IVIg dose at the time of first SCIg administration (‘switch’) and at 3 (‘3 months’) and 6 months (‘6 months’) during therapy with SCIg (see Supplemental Figure 1 for study design). The Inflammatory Neuropathy Cause and Treatment disability score (INCAT) was used to measure CIDP-related disability.^7^ The Inflammatory Rasch-Built Overall Disability Scale (I-RODS) was applied to assess activity and limitations on social participation and raw values (0–48), as well as the converted centile score (0–100) were calculated.^2,8^ Overall muscle strength was measured by Medical Research Council (MRC) sum score for eight muscle groups (including shoulder abduction, elbow flexion, wrist extension, index finger abduction, hip flexion, knee extension, foot dorsiflexion, and great toe dorsiflexion; range of total score: 0–80).^2,9^ Grip strength for both hands was assessed with the Martin Vigorimeter (Martin, Tuttlingen, Germany) by using the mean of three measurements of maximum grip strength.^10^ Gait impairment was evaluated by the Timed 100-Meter Walk Test and by establishing the mean of two passages of the Timed 25-Foot Walk Test.^11^ Systemic and local reactions, dose of intravenous or subcutaneous immunoglobulin, as well as patient preferences were recorded. Using a numeric rating scale ranging from 0 (totally dissatisfied) to 10 (totally satisfied), treatment satisfaction was evaluated at every time point.

### Statistical analysis

All statistical analyses were performed using GraphPad Prism 9.0 (GraphPad Software, San Diego, CA, USA). Normal distribution of values was evaluated by D’Agostino-Pearson omnibus test and depending on the results, the nature of the investigated variable and the number of comparisons, unpaired *t* test, Mann–Whitney *U* test, Fisher’s exact test, one-way analysis of variance with Bonferroni *post hoc* test or Kruskal–Wallis test with Dunn’s test for multiple comparisons were performed, respectively. Results were considered statistically significant at *p* ⩽ 0.05.

## Results

### Preferences of CIDP patients regarding switch from IVIg to SCIg (IgPro20)

After approval of IgPro20, 102 potential patients with CIDP seen at the Department of Neurology of Hannover Medical School were offered a treatment change from IVIg to SCIg. Forty-one patients (40%) preferred to switch to SCIg. The most frequently cited reasons for therapy change were ‘avoiding hospitalization’ (32%), ‘convenience/better integration of treatment into everyday life’ (29%) and ‘achieving greater autonomy’ (29%). Sixty-one patients refused to switch from IVIg to SCIg, mainly due to ‘feeling safer in the hospital’ (36%), ‘lack of confidence in SCIg administration’ (18%) and ‘feeling of clinical instability’ (16%; Table 1).

**Table 1.** Patient preferences.

CIDP patients evaluated for switch from IVIg to SCIg (*n* = 102)		
Yes (*n* = 41; 40%)	Reasons for switch to SCIg	*n*/41 (%)
	Avoiding hospitalization	13 (32)
	Convenience/better integration into everyday life	12 (29)
	Achieving greater autonomy	12 (29)
	Preventing fluctuation of therapeutic effect	9 (22)
	Avoiding journey to hospital	8 (20)
	Better compatibility with work	6 (15)
	Avoiding side effects of IVIg	2 (5)
	Difficult vein conditions	2 (5)
No (*n* = 61; 60%)	Reasons against switch to SCIg	*n*/61 (%)
	Feeling safer in hospital	22 (36)
	Lack of confidence in SCIg administration	11 (18)
	Feeling of clinical instability	10 (16)
	No wish to change due to current clinical stability	6 (10)
	Impaired upper extremity motor function	5 (8)
	Language barrier	4 (7)
	Fear of adverse effects in domestic environment	3 (5)
	Fear of pricking oneself	3 (5)
	Cognitive impairment	3 (5)
	Adjustment too inconvenient	1 (2)
	Fear of consequences for work	1 (2)
	Scepticism about mode of therapy	1 (2)

Evaluation of patient preferences in a cohort of 102 patients with CIDP. Reasons for (‘Yes’) and against (‘No’) a transition from IVIg to SCIg are given. Multiple answers were possible.

CIDP, chronic inflammatory demyelinating polyneuropathy; IVIg, intravenous immunoglobulin; SCIg, subcutaneous immunoglobulin.

### Patient characteristics

A total of 41 (32 male and 9 female) patients with CIDP who decided to switch from IVIg to SCIg were included in the analysis. The median age of patients at the time of transition to SCIg was 60 years [51–67 interquartile range (IQR)] with a median disease duration of 30 months (10.5–59.5 IQR). The median duration of previous IVIg therapy was 20 months (6.5–54.5 IQR) and the INCAT score at baseline was 3 (2–4; median and IQR). In comparison with patients who decided against a transition to SCIg and remained under IVIg treatment, patients who switched were younger, had a higher previous IVIg dose and a lower INCAT score, and thus, less clinical disability. No significant differences were found regarding disease duration and duration of previous IVIg treatment (see Table 2 for further baseline characteristics).

**Table 2.** Patient baseline characteristics.

	Yes (*n* = 41)	No (*n* = 61)	*p*
Sex, *n* (%)			
Female	9 (22)	24 (39)	0.085
Male	32 (78)	37 (61)	
Age at the time of evaluation of switch to SCIg (years), median (IQR)	60 (51–67)	67 (58–77)	0.008
Disease duration (months), median (IQR)	30 (10.5–59.5)	32 (9–85.5)	0.811
Duration of previous IVIg treatment (months), median (IQR)	20 (6.5–54.5)	24 (6–67.5)	0.596
Previous IVIg interval (weeks), median (IQR)	4 (4–6)	5 (4–6)	0.005
Weekly immunoglobulin dose (g), mean (SD)	21.5 (7.7)	16.6 (5.5)	<0.001
CIDP diagnostic criteria, *n* (%)			
Definite	39 (95)	48 (79)	0.024
Probable	2 (5)	13 (21)	
Baseline INCAT, median (IQR)	3 (2–4)	4 (3–5.5)	<0.001

Baseline characteristics of patients who decided for and against a change from IVIg to SCIg. Patients who switched to SCIg were younger, had a higher previous weekly IVIg dose and had a lower INCAT compared with patients who preferred to continue IVIg treatment.

CIDP, chronic inflammatory demyelinating polyneuropathy; INCAT, Inflammatory Neuropathy Cause And Treatment disability score; IQR, interquartile range; IVIg, intravenous immunoglobulin; SCIg, subcutaneous immunoglobulin; SD, standard deviation.

### Clinical stability after dose-equivalent switch from IVIg to SCIg

**Figure 1.**
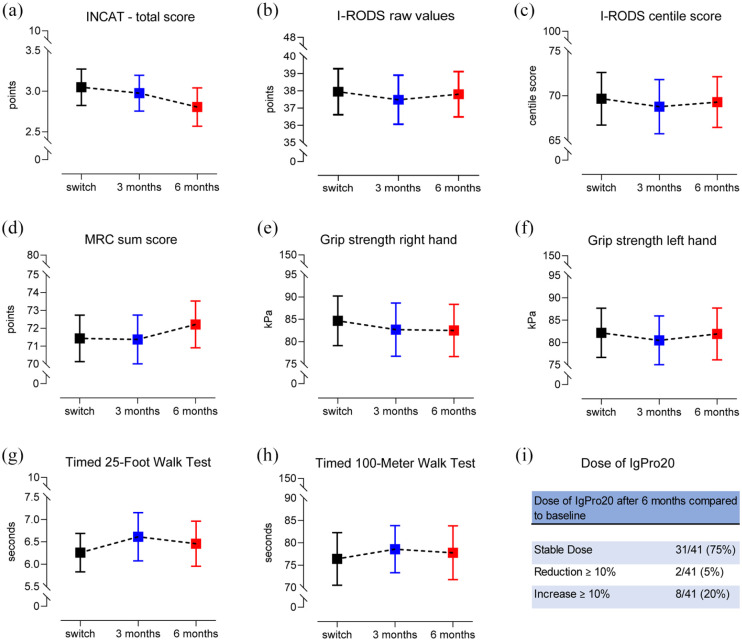
Clinical stability after switch from IVIg to SCIg (IgPro20). Clinical stability between the different time points ‘switch’ (last dose of IVIg), ‘3 months’ (3 months after transition to SCIg) and ‘6 months’ (6 months after transition to SCIg; a–h). No significant differences between the time points were detected for all outcome parameters. The majority of patients remained on a stable dose of IgPro20 during 6 months after switch to SCIg (i). IgPro20, an approved subcutaneous immunoglobulin; INCAT, Inflammatory Neuropathy Cause and Treatment disability score; I-RODS, Inflammatory Rasch-Built Overall Disability Scale; IVIg, intravenous immunoglobulin; MRC, Medical Research Council; SCIg, subcutaneous immunoglobulin.

The INCAT score, as a tool to measure clinical disability in patients with CIDP, remained stable after the transition from IVIg to SCIg [Figure 1(a)]. In addition, no significant changes were observed for the patient-reported I-RODS scale, capturing clinically meaningful impairment, after switch to subcutaneous IgPro20 [Figure 1(b) and (c)]. Clinical stability was further demonstrated by evaluation of the MRC sum score which revealed no significant differences of muscle strength between the investigated time points [Figure 1(d)]. In addition, measurement of maximum grip strength for both hands by Martin Vigorimeter showed stable values after the therapy transition to IgPro20 [Figure 1(e) and (f)]. Moreover, the Timed 25-Foot Walk Test as well as the Timed 100-Meter Walk Test revealed no significant changes after switch from IVIg and SCIg treatment [Figure 1(g) and (h)].

In line with these results, the dose of subcutaneous IgPro20 remained stable in 75% of patients and could be reduced by ⩾10% in 5% of patients after 6 months of treatment with SCIg. In 20% of patients, the dose of IgPro20 was increased ⩾10% during 6 months of SCIg treatment in comparison with the time point of transition [Figure 1(i)]. Three patients received IVIg as rescue medication due to a temporary clinical deterioration. No patient discontinued the therapy with IgPro20 during the 6-month observation period.

### Patient treatment satisfaction and preferences

Treatment satisfaction remained stable after the switch from IVIg (8.1 ± 1.8; mean ± standard deviation) to SCIg after 3 (8.1 ± 1.5) and after 6 months (7.7 ± 2.0).

Treatment preferences were recorded after 6 months of IgPro20 therapy. A total of 88% (36/41) of patients preferred treatment with subcutaneous IgPro20 to IVIg treatment. Only 7% (3/41) favoured treatment with IVIg over SCIg, while 5% (2/41) of patients were undecided (Table 3).

**Table 3.** Treatment satisfaction and preferences.

	NRS (0–10), mean (SD)
Treatment satisfaction	
Switch: satisfaction with IVIg	8.1 (1.8)
3 Months: satisfaction with SCIg	8.1 (1.5)
6 Months: satisfaction with SCIg	7.7 (2.0)
	*n*/41 (%)
Treatment preference after 6 months of SCIg	
Preference of SCIg	36 (88)
Preference of IVIg	3 (7)
Undecided	2 (5)

No significant differences were observed for treatment satisfaction between the time points. Treatment preferences queried after 6 months of therapy with IgPro20 showed that the majority (36/41; 88%) of patients preferred SCIg over IVIg.

IVIg, intravenous immunoglobulin; NRS, numeric rating scale; SCIg, subcutaneous immunoglobulin; SD, standard deviation.

### Systemic and local reactions

While 56% (23/41) and thus the majority of patients reported systemic reactions during IVIg therapy, only 29% (12/41) of patients reported systemic reactions after 6 months of SCIg treatment, with headache and efflorescences being the most frequent symptoms. The proportion of patients with local reactions increased from 12% (5/41) during treatment with IVIg to 39% (16/41) of patients after 3 months and 27% (11/41) after 6 months after switch to SCIg. Infusion-site pruritus, swelling, hardening and erythema were the most frequently reported local reactions (Supplemental Table 1). Local reactions were mild, temporary, and did not lead to discontinuation of SCIg treatment.

## Discussion

Our data show that under real-world conditions, approximately only half of the eligible CIDP patients wanted to switch to SCIg, suggesting that IVIg still represents an important treatment modality. Patients who decided for a transition from IVIg to SCIg were younger, received a higher IVIg dose and had a lower INCAT score and hence less disability compared with patients who preferred to resume IVIg therapy.

The dose-equivalent transition from IVIg to subcutaneous IgPro20 resulted in overall clinical stability in CIDP patients regarding disability, general activity and social participation, grip and muscle strength, as well as gait impairment. Accordingly, a majority of 80% of patients remained on a stable or reduced dose of IgPro20 after 6 months of SCIg therapy. It is noteworthy, that although treatment satisfaction remained unchanged between IVIg and SCIg therapy, 88% of patients preferred treatment with subcutaneous IgPro20 over IVIg 6 months after switch to SCIg. The transition of IVIg to SCIg resulted in a change of reaction profile with fewer patients reporting systemic reactions and more patients describing local reactions under treatment with SCIg compared with IVIg.

Our results underline the efficacy of SCIg in CIDP as reported in previous publications, including the PATH and its extension study.^2–5,12^ Here, we were able to demonstrate efficacy, tolerability, satisfaction, and preference of SCIG in a cohort of CIDP patients under real-world conditions with a comprehensive set of outcome parameters. By avoiding a study-related predefinition of patient cohorts or treatment regimens, these data are highly relevant for future therapy management of patients with CIDP in daily clinical practice. However, the results of our study were obtained after an observation period of 6 months and thus represent a short-term outcome. Further real-life studies are needed to examine the long-term outcomes after switch from IVIg to SCIg.

Based on our results, we conclude that SCIg represents an effective, safe, and preferred treatment option for CIDP patients.

## Supplemental Material

sj-tif-1-tan-10.1177_17562864211009100 – Supplemental material for Switch from intravenous to subcutaneous immunoglobulin IgPro20 in CIDP patients: a prospective observational study under real-world conditions Click here for additional data file. Supplemental material, sj-tif-1-tan-10.1177_17562864211009100 for Switch from intravenous to subcutaneous immunoglobulin IgPro20 in CIDP patients: a prospective observational study under real-world conditions by Stefan Gingele, Moritz Koch, Anna Christina Saparilla, Gudrun M. Körner, Jarle von Hörsten, Marina Gingele, Tabea Seeliger, Franz Felix Konen, Martin W. Hümmert, Alexandra Neyazi, Martin Stangel and Thomas Skripuletz in Therapeutic Advances in Neurological Disorders

sj-xlsx-2-tan-10.1177_17562864211009100 – Supplemental material for Switch from intravenous to subcutaneous immunoglobulin IgPro20 in CIDP patients: a prospective observational study under real-world conditions Click here for additional data file. Supplemental material, sj-xlsx-2-tan-10.1177_17562864211009100 for Switch from intravenous to subcutaneous immunoglobulin IgPro20 in CIDP patients: a prospective observational study under real-world conditions by Stefan Gingele, Moritz Koch, Anna Christina Saparilla, Gudrun M. Körner, Jarle von Hörsten, Marina Gingele, Tabea Seeliger, Franz Felix Konen, Martin W. Hümmert, Alexandra Neyazi, Martin Stangel and Thomas Skripuletz in Therapeutic Advances in Neurological Disorders
