# Supplementary figures and images for: Switch from intravenous to subcutaneous immunoglobulin IgPro20 in CIDP patients: a prospective observational study under real-world conditions
Source: Ther Adv Neurol Disord. 2021 Apr 16;14:17562864211009100. doi: 10.1177/17562864211009100 (PMC8053839; doi:10.1177/17562864211009100)

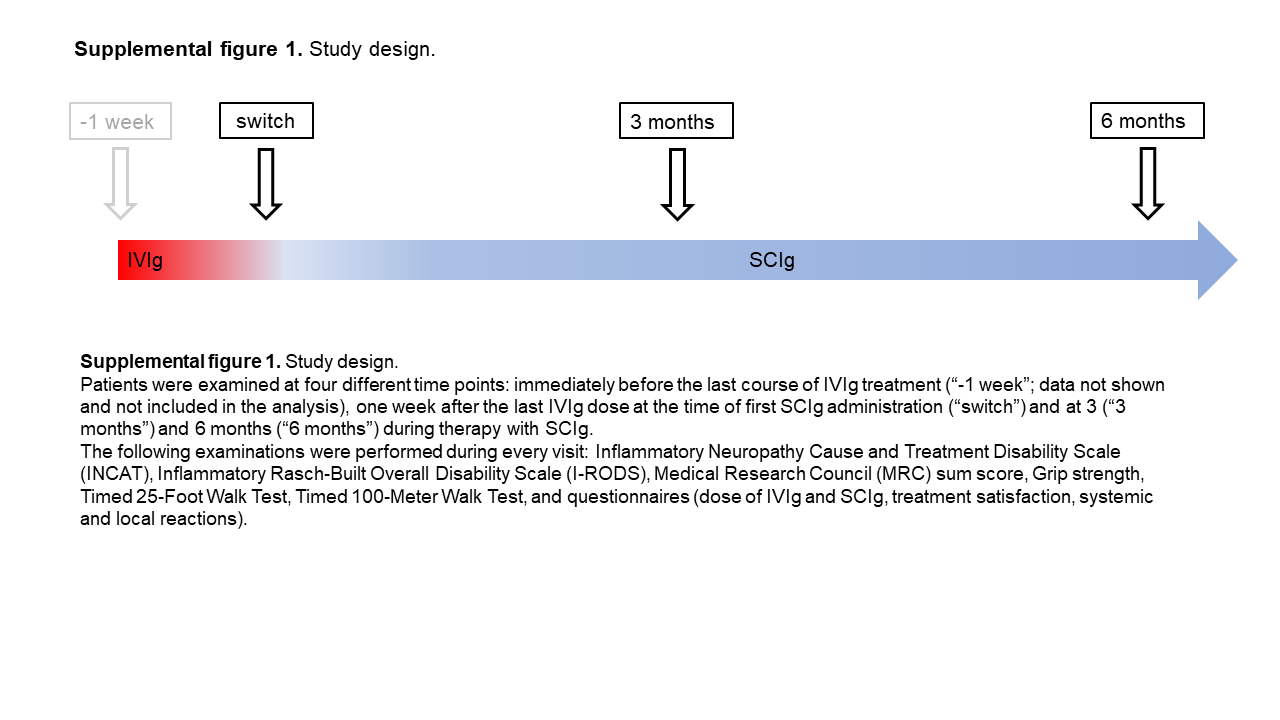

Supplement: sj-tif-1-tan-10.1177_17562864211009100 – Supplemental material for Switch from intravenous to subcutaneous immunoglobulin IgPro20 in CIDP patients: a prospective observational study under real-world conditions [file sj-tif-1-tan-10.1177_17562864211009100.tif]
